# Anti-inflammatory and Tendon-Protective Effects of Adipose Stem Cell-Derived Exosomes with Concomitant Use of Glucocorticoids

**DOI:** 10.1155/2022/1455226

**Published:** 2022-05-20

**Authors:** Xuancheng Zhang, Ang Li, Kang Han, He Zhang, Xiaoqiao Huangfu, Jinghuan Huang, Jia Jiang, Jinzhong Zhao

**Affiliations:** ^1^Department of Sports Medicine, Shanghai Jiao tong University Affiliated Sixth People's Hospital, Shanghai, China; ^2^Fengfeng General Hospital of North China Medical and Health Group, Hebei, China

## Abstract

Glucocorticoid (GC) injections are commonly used in clinical practice to relieve pain and improve function in patients with multiple shoulder disabilities but cause detrimental effects on rotator cuff tendons. Adipose stem cell-derived exosomes (ASC-Exos) reportedly recover impaired tendon matrix metabolism by maintaining tissue homeostasis. However, it is unclear whether additional treatment with ASC-Exos overrides the detrimental effects of GCs without interfering with their anti-inflammatory effects. Thus, we aimed to investigate the anti-inflammatory effect of ASC-Exos with GCs and protective effect of ASC-Exos against GC-induced detriments. The present study comprised in vitro and in vivo studies. In vitro inflammatory analysis revealed that ASC-Exos exerted a synergic anti-inflammatory effect with GCs by significantly decreasing secretion of proinflammatory cytokines by RAW cells and increasing secretion of anti-inflammatory cytokines. In vitro cytoprotective analysis showed that ASC-Exos overrode GC-induced detrimental effects on tenocytes by significantly improving GC-suppressed cellular proliferation, migration, and transcription of tenocytic matrix molecules and degradative enzyme inhibitors and significantly decreasing GC-induced cell senescence, apoptosis, and transcription of ROS and tenocytic degradative enzymes. In vivo studies revealed that additional ASC-Exo injection restored impairments in histological and biomechanical properties owing to GC administration. Collectively, these results suggest that ASC-Exos exert a stronger anti-inflammatory effect in combination with GCs, overriding their detrimental effects on rotator cuff tendons.

## 1. Introduction

Shoulder disability embodies multiple pathologic conditions, including rotator cuff tear, frozen shoulder, calcified tendinitis, and other tendinopathies [1, 2]. Irrespective of the cause, shoulder disability leads to shoulder pain and limited range of motion. The mainstay of treatment is conservative therapy, including rest, activity modification, physical therapy, and local injection of nonsteroidal anti-inflammatory drugs or corticosteroids [2–7]. The most common of these conservative therapies is glucocorticoid (GC) injection [3–5].

Single or repeated GC injections reportedly significantly reduce shoulder pain and improve shoulder range of motion in athletes with chronic pain [8]. The underlying mechanism is due to the anti-inflammatory effect of GCs [9, 10]. Shoulder injury is followed by a massive infiltration of inflammatory cells and secretion of several upstream inflammatory cytokines such as TNF-*α* and IL-1, which triggers a proinflammatory reaction and ultimately induces the formation of pain-related neuropeptides [11–14]. GCs achieve their therapeutic effect by inhibiting infiltration of inflammatory cells and secretion of upstream proinflammatory factors [9, 10, 14].

Although GCs have potent anti-inflammatory and analgesic effects, they also have important adverse effects. There is increasing concern that GC injections may accelerate tendon degeneration [2, 6, 15]. Tendon rupture after GC injections is common in both the upper and lower extremities in clinical practice [16, 17]. Although it is unclear to what extent such ruptures are attributable to GC injections or the progression of tendinopathy itself, several studies have confirmed that GC injections are correlated with tendon detriments [2, 6, 15]. Haraldsson et al. showed a significant decrease in the tensile strength of isolated rat-tail collagen fascicles at 3 and 7 days after GC treatment compared with the saline-treated group [6]. Mikolyzk et al. and Maman et al. reported the detrimental effect of single and repeated GC injections on both injured and intact rotator cuff tendons [2, 15]. The adverse effects of GC injections on the tendon are believed to be due to the negative effect of GCs on tendon metabolism homeostasis in combination with decreased cell proliferation and increased cell senescence, apoptosis, and destruction of normal collagen organization [2, 6, 7, 15, 18]. Therefore, concomitant treatment with a bioactive substance that overrides the detrimental effects of GCs on cellular metabolism homeostasis may be a practical strategy to minimize the adverse effects of GCs [7, 18]. However, despite considerable research, there is still no efficacious means by which to eliminate the undesirable effects of GCs while retaining their anti-inflammatory effects.

Exosomes are nanosized membrane vesicles that contain proteins, lipids, and nucleic acid and play an important role in the regulation of cellular physiological activities [19–25]. They have attracted increasing interest and have been widely studied in tissue-engineering fields in recent years [19–25]. In our previous studies, we found that adipose stem cell-derived exosomes (ASC-Exos) were able to recover the impaired matrix metabolism of torn rotator cuff tendons by maintaining tissue homeostasis. Drawing on this, we inferred that additional treatment with ASC-Exos may override the detrimental effects of GCs on tenocytes without interfering with their anti-inflammatory effects.

The present study was aimed at (1) investigating the anti-inflammatory and cytoprotective effects of ASC-Exos on RAW cells and tenocytes, respectively, with concomitant treatment of GCs in vitro and (2) evaluating the effect of ASC-Exos on injured-intact tendons with concomitant GC injection in vivo using a rat model of chronic rotator cuff tear. Our hypothesis was that the additional ASC-Exo treatment would exert a synergic anti-inflammatory effect with GCs and cytoprotective effect against their adverse effects and would improve both the histological and biomechanical properties of injured-intact rotator cuffs simultaneously injected with GCs in vivo.

## 2. Methods

### 2.1. Isolation and Characterization of ASC-Exos.

ASC isolation and culture and ASC-Exo isolation were performed as described in previous studies [26, 27]. Briefly, cells were cultured in serum-free medium (OriCell; Cyagen Biosciences, Santa Clara, CA, USA) and incubated at 37°C in a humidified atmosphere with 5% CO_2_. Cells from the second and third passages were used for isolation of exosomes. After cell confluency reached 70% -80%, cells were rinsed with phosphate-buffered saline (PBS) and cultured in fresh serum-free medium (25 mL/dish) for 48 hours to collect the supernatant. The medium was centrifuged at 300 × *g* for 10 minutes and 2000 × *g* for 10 minutes to remove cells and further centrifuged at 10,000 × *g* for 30 minutes to remove cell debris. Next, the medium was ultracentrifuged at 100,000 × *g* for 70 minutes to obtain ASC-Exo pellets at the bottom of centrifuge tubes. ASC-Exos were purified after further resuspension and centrifugation to eliminate contaminating proteins. The morphology of ASC-Exos was observed using transmission electron microscopy (TEM; H-7650; Hitachi High-Technologies Corporation, Tokyo, Japan). The particle size distribution was evaluated using nanoparticle tracking analysis (NTA; ZetaView PMX 120, Particle Metrix, Meerbusch, Germany). Expression of exosomal surface markers CD9, CD63, and TSG-101 was detected using western-blotting. Briefly, protein extracts were separated using sodium dodecyl sulfate-polyacrylamide gel electrophoresis and blotted onto a polyvinylidene difluoride membrane (Merck-Millipore, Readington, NJ, USA). Subsequently, membranes were blocked with 5% nonfat dried milk in Tris-buffered saline (10 mM Tris-HCl pH 7.5, 150 mM NaCl, and 0.1% Tween-20) and incubated overnight with primary antibodies, followed by incubation with horseradish-peroxidase labeled secondary antibodies (Cell Signaling Technology, Danvers, MA, USA).

### 2.2. In Vitro Studies

#### 2.2.1. Inflammation Analysis

*(1) Interventions and Study Groups*. RAW cells (RAW 264.7), a rat macrophage cell line, were used for inflammation analysis in the present study. In accordance with previous studies [12, 28], cells were pretreated with complete culture medium [Dulbecco's Modified Eagle's Medium (DMEM) supplemented with 10% fetal bovine serum (FBS), 1% penicillin, 10 ng/mL macrophage colony-stimulating factor, and 100 ng/mL lipopolysaccharide] for 24 hours to allow attachment. Subsequently, cells were treated with saline (control group), 1 *μ*M dexamethasone (GC group), or 1 *μ*M dexamethasone and 10^11^ pellets/mL ASC-Exos (GC+ASC-Exo group) and further evaluated.

### 2.3. Cell Proliferation

After seeding in 96-well plates at a density of 1 × 10^3^ cells/well, cells were incubated for 24 hours (to allow attachment) and then treated with saline, dexamethasone, or dexamethasone and ASC-Exos (dexamethasone+ASC-Exos). Cell proliferation was evaluated at 12, 24, and 48 hours using a Cell Counting Kit 8 (CCK-8) assay in accordance with the manufacturer's instructions (Dojindo Molecular Technologies, Kumamoto, Japan). Briefly, at 12, 24, and 48 hours, CCK-8 solution was added to wells and incubated for an additional 1 hour at 37°C. The absorbance of each well was measured at 450 nm using a spectrophotometric microplate reader (Bio-Rad 680, Bio-Rad, Hercules, CA, USA). Cell proliferation was calculated as the optical density of the tested well minus the absorbance of a blank well.

### 2.4. Cell Migration

After seeding in the upper chamber of 96-well Transwell plates (Corning, Corning, NY, USA) at a density of 1 × 10^6^ cells/well, cells were incubated for 24 hours to allow attachment. Next, saline, dexamethasone, or dexamethasone+ASC-Exos was added to the lower chamber. After incubation for an additional 24 hours, cell migration was evaluated in accordance with the manufacturer's instructions. Briefly, cells from the upper surface of the filter membranes were removed with a cotton swab. Cells that migrated to the lower surface of the filter membrane were fixed in 4% formaldehyde for 5 minutes and then stained with 0.5% crystal violet for 5 minutes. Migration activity was evaluated by observing stained cells under an optical microscope.

### 2.5. Enzyme Linked Immunosorbent Assay (ELISA)

After seeding in 96-well plates at a density of 1 × 10^5^ cells/well, cells were incubated for 24 hours (to allow attachment) and then treated with saline, dexamethasone, or dexamethasone+ASC-Exos. Levels of secreted inflammation-related cytokines TNF-*α*, IL-1*α*, IL-1*β*, IL-4, and IL-10 were evaluated using an ELISA kit in accordance with the manufacturer's instructions (R&D Systems, Minneapolis, MN, USA). Briefly, the culture medium of cells incubated for an additional 96 hours was centrifugated to obtain the supernatant, which was then incubated with specific antibodies for 2 hours at 37°C in a 96-well plate. After washing, each well was incubated with the corresponding conjugate for 1 hour, and substrate solution was added to develop the color in the dark. The absorbance of each well at 450 nm was determined using a microplate reader (Multiskan MK3; ThermoFisher Scientific, Waltham, MA, USA).

### 2.6. Cytoprotective Analysis

#### 2.6.1. Isolation and Culture of Rat Tenocytes

Rotator cuffs were harvested from adult rats and washed several times with PBS. The tendons were then cut into 1 mm × 1 mm × 1 mm fragments and digested using trypsin and collagenase. After sufficient digestion, the tissues were centrifuged and the supernatant was removed. Subsequently, tissues were transferred into complete medium and placed in an incubator. The medium was changed every 3 days, and any obvious tissue masses were discarded. Cell passaging started when cells reached 80%–90% confluency, and cells from the third to fifth passages were used in the present study.

### 2.7. Interventions and Study Groups

After incubation in complete DMEM supplemented with 10% FBS and antibiotic solution for 24 hours to allow attachment, cells were treated with saline (control group), 1 *μ*M dexamethasone (GCs group), or 1 *μ*M dexamethasone and 10^11^ pellets/mL of ASC-Exos (GCs+ASC-Exo group) and further evaluated.

### 2.8. Cell Proliferation

After seeding in 96-well plates at a density of 1 × 10^3^ cells/well, cells were incubated for 24 hours to allow attachment and then treated with saline, dexamethasone, or dexamethasone+ASC-Exos. Cell proliferation was evaluated at 12, 24, and 72 hours using a CCK-8 assay kit as described above.

### 2.9. Cell Migration

After seeding in the upper chamber of a 96-well Transwell plate at a density of 1 × 10^4^ cells/well, cells were incubated for 24 hours to allow attachment. Saline, dexamethasone, or dexamethasone+ASC-Exo was then added to the lower chamber. Cell migration was evaluated as described above.

### 2.10. Cell Senescence

After seeding in a 6-well plate at a density of 1 × 10^4^ cells/well, cells were incubated for 24 hours to allow attachment and then treated with saline, dexamethasone, or dexamethasone+ASC-Exos. The senescence assay was performed using a senescence *β*-galactosidase staining kit in accordance with the manufacturer's instructions (Solarbio Science and Technology, Beijing, China). Briefly, after incubation for another 24 hours, 1 mL/well of senescence *β*-galactosidase staining solution was added to each well and incubated for 15 minutes at 37°C. Senescent cells were stained with the prepared dye and fixed at 37°C overnight. Cell senescence was evaluated by calculating the intensity of positively stained cells under an optical microscope using ImageJ software (National Institutes of Health).

### 2.11. Cell Apoptosis

After seeding in a 96-well plate at a density of 1 × 10^4^ cells/well, cells were incubated for 24 hours to allow attachment before being treated with saline, dexamethasone, or dexamethasone+ASC-Exos. The apoptotic assay was performed using a TUNEL apoptosis assay kit in accordance with the manufacturer's instructions (Solarbio Science and Technology, Beijing, China). Briefly, after incubation for another 24 hours, 50 *μ*L/well of TUNEL solution was added to each well and incubated for a further 1 hour at 37°C. Apoptotic cells were stained with reaction buffer, and their fluorescence intensity was evaluated using a flow cytometer with an FL3 channel.

### 2.12. Real-Time Polymerase Chain Reaction (RT-PCR)

After seeding in a 6-well plate at a density of 1 × 10^6^ cells/well, cells were incubated for 24 hours to allow attachment. Cells were then treated with saline, dexamethasone, or dexamethasone+ASC-Exos and incubated for a further 72 hours. Relative mRNA transcription levels of ROS, MMP-2, MMP-9, MMP-13, TIMP-1, TIMP-3, decorin, biglycan, and type I and type III collagens were evaluated as previously described [27]. Briefly, TRIzol reagent (Invitrogen, Carlsbad, CA, USA) was used to extract total RNA from cells. Reverse transcription was performed using Reverse Transcription Supermix (Bio-Rad Laboratories). RT-PCR was conducted using a CFX Connect Real-Time PCR System (Bio-Rad) with Universal SYBR Green Supermix (Bio-Rad). Relative mRNA transcription levels of genes were calculated using the 2-*ΔΔ*Ct method (primer sequences for mRNAs used in the present study are provided in the supplementary materials (available here)). GAPDH was used as a housekeeping gene to normalize expression of genes of interest in the present study.

### 2.13. In Vivo Studies

#### 2.13.1. Power Analysis and Study Groups

The animal experimental protocol was approved by the Animal Ethical and Welfare of Shanghai Jiaotong University Department.

A power analysis was performed with *α* = 0.05, 1 − *β* = 0.8, and an assumed dropout rate of 25% to calculate the sample size required for animal experiments. A sample size of eight shoulders was required to detect a significant difference in biomechanical analysis of ultimate load to failure. In addition, we allocated four shoulders for histological analysis in each group. Thus, a total of 36 shoulders (18 male Sprague Dawley rats aged 12 weeks) were included in the present study and divided into the following three groups: saline injection group (control group), dexamethasone injection group (GCs group), and dexamethasone+ASC-Exo injection group (GC+ASC-Exo group). Each shoulder was randomly chosen to receive an injection of saline, dexamethasone, or dexamethasone+ASC-Exos.

### 2.14. Establishment of Chronic Rotator Cuff Injured-Intact Model and Interventions

After induction of general anesthesia, a 1 cm longitudinal skin incision was made over the lateral shoulder joint under sterile conditions. The supra-infraspinatus tendon was exposed by splitting the deltoid muscle. The rotator cuff injured-intact model was established by completely cutting the supraspinatus tendon from the greater tubercle to create a full-thickness injury while leaving the infraspinatus tendon intact; this was left for 6 weeks to establish a chronic injured-intact model that simulated the clinical setting [29, 30]. The muscle, subcutaneous tissue, and skin were closed as separate layers.

**Figure 1:**
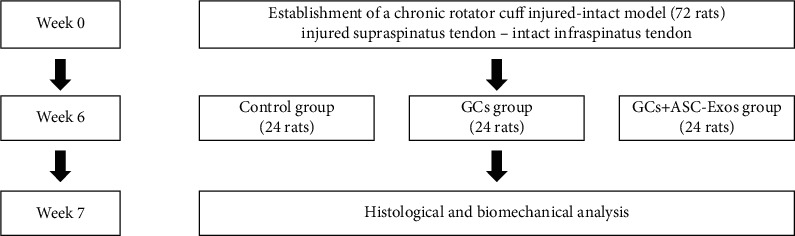
Animal model establishment and study groups.

After 6 weeks, a single dose of saline, dexamethasone (0.1 mg/kg), or dexamethasone (0.1 mg/kg)+ASC-Exos (10^11^ pellets/mL) of the same volume was injected into the subacromial space in each shoulder. At 1 week after injection, all rats were euthanized by CO_2_ inhalation. The supra-infraspinatus-humerus complex was carefully harvested, and histological and biomechanical analysis were performed for each harvested sample (Figure 1).

### 2.15. Histological Analysis

Samples were fixed in 4% buffered paraformaldehyde for 24 hours and embedded in paraffin. Consecutive 3 *μ*m-thick sections were cut parallel to the long axis of the supra-infraspinatus tendon in the coronal plane.

To evaluate their general appearance, sections were stained with hematoxylin and eosin (HE). To evaluate collagen degeneration, sections were stained with type I and III collagen antibodies. To evaluate fatty infiltration, sections were stained with oil red O.

Images of the entire section of the supra-infraspinatus were acquired by digital slide scanning (Pannoramic MIDI; 3DHISTECH Ltd.). Intensities of type I and type III collagen and fatty infiltration were quantitatively measured using ImageJ software as previously described [26].

Semiquantitative analysis was also performed as previously described to comprehensively evaluate the histological properties of each tendon (Table 1, [27]).

### 2.16. Biomechanical Analysis

Before biomechanical testing, dissections were performed to separate the supra-infraspinatus-humerus complex into two parts: the isolated supraspinatus and the infraspinatus-humerus complex. The cross-sectional areas of both the supra- and infraspinatus tendons were measured using digital calipers. A custom-designed uniaxial testing machine (Instron 5569, USA) was used to perform the biomechanical analysis.

**Figure 2:**
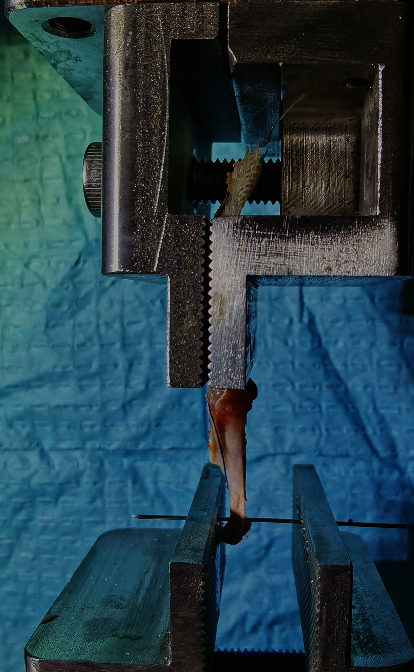
A representative picture of biomechanical analysis.

To fix the isolated supraspinatus, the muscular end was gripped in a serrated clamp to prevent slippage, and the tendinous end was cross-sutured using No. 2 Orthocord suture (DePuy Mitek) and then tied to the sensor of the device. To fix the infraspinatus-humerus complex, the humerus was mounted using a 0.8 mm K wire, and the muscular end was gripped in a serrated clamp connected to the sensor system (Figure 2).

After preloading for 5 minutes using a 0.1 N tensile force, the sample received 10 cycles of loading ranging from 0.1 N to 1 N to minimize the viscoelastic effects. Immediately after preconditioning, the load-to-failure test started with uniaxial tension at 1 mm/minute. The ultimate load to failure was defined as the first significant decrease in the load-displacement curve, and the stress was calculated by dividing the ultimate load to failure by the initial cross-sectional area. The mode of failure was also recorded.

### 2.17. Statistical Analysis

All data are expressed as mean ± SD. Statistical analysis was performed using SPSS software (version 15, SPSS). One-way analysis of variance with post hoc testing using the Bonferroni method was carried out. Significance was set at *P* < 0.05.

## 3. Results

### 3.1. Characterization of ASC-Exos

**Figure 3:**
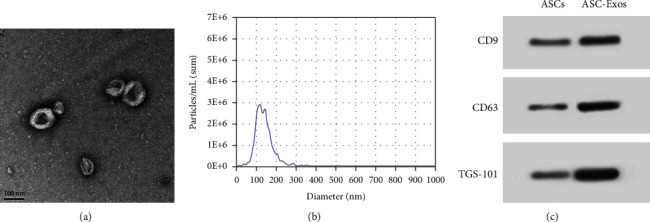
Identification of ASC-Exos. (a) The morphology of ASC-Exos was observed via TEM. (b) The particle size distribution was evaluated using NTA. (c) Exosome surface markers including CD9, CD63, and TSG-101 were evaluated using western-blotting.

TEM and NTA revealed spherical vesicles with a particle size distribution of 50 to 150 nm. Western-blotting analysis showed that these vesicles had strong surface expression of exosomal markers, including CD9, CD63, and TSG-101 (Figure 3).

### 3.2. Inflammation Analysis

#### 3.2.1. Cell Proliferation

Compared with the control group, dexamethasone treatment significantly decreased the proliferation intensity of rat RAW cells to 0.89-, 0936-, and 0.849-fold at 12, 24, and 48 hours, respectively.

**Figure 4:**
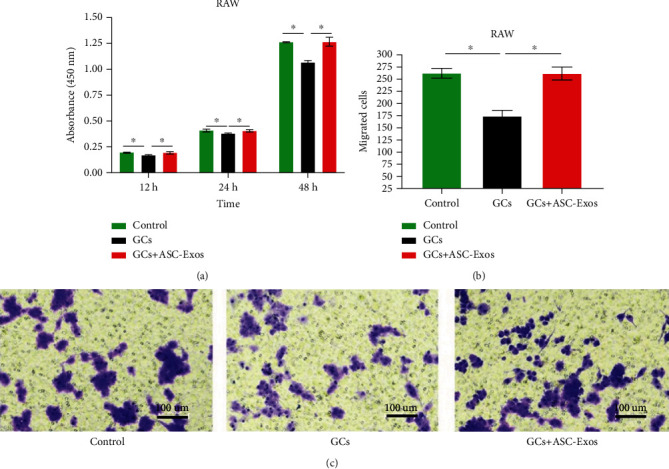
(a) Statistical comparison of rat RAW cell proliferation intensity in the saline-treated group (control), GC-treated group (GCs), and GC+ASC-Exo-treated group (GCs+ASC-Exos). (b) Statistical comparison of rat RAW cells migration activity in the saline-treated group (control), GC-treated group (GCs), and GC+ASC-Exo-treated group (GCs+ASC-Exos). (c) Representative figures of rat RAW cells migration activity in the saline-treated group (control), GC-treated group (GCs), and GC+ASC-Exo-treated group (GCs+ASC-Exos). Data are expressed as mean ± SD. ^∗^indicates significant differences, *P* < 0.05.

To our surprise, additional treatment with ASC-Exos did not further decrease the proliferation intensity but overrode the detrimental effect of dexamethasone on the proliferation of RAW cells, with the GC+ASC-Exo group showing no significant differences in proliferation intensity compared with the control group (Figure 4(a)).

### 3.3. Cell Migration

Dexamethasone treatment significantly decreased the migration of rat RAW cells to 0.66-fold at 24 hours, compared with the control group.

Consistent with cell proliferation results, additional treatment with ASC-Exos did not further decrease cell migration but overrode the detrimental effect of dexamethasone on the migration of RAW cells, with the GC+ASC-Exo group showing no significant differences in migration compared with the control group (Figures 4(b) and 4(c)).

### 3.4. Secretion of Inflammatory-Related Cytokines

#### 3.4.1. Proinflammatory Cytokines

Dexamethasone treatment significantly downregulated secretion of TNF-*α*, IL-1*α*, and IL-1*β* from rat RAW cells to 0.754-, 0.87-, and 0.59-fold, respectively, compared with the control group.

In contrast to cell proliferation and migration results, additional treatment with ASC-Exos further significantly downregulated secretion of TNF-*α*, IL-1*α*, and IL-1*β* to 0.62-, 0.77-, and 0.438-fold, respectively, compared with the control group.

**Figure 5:**
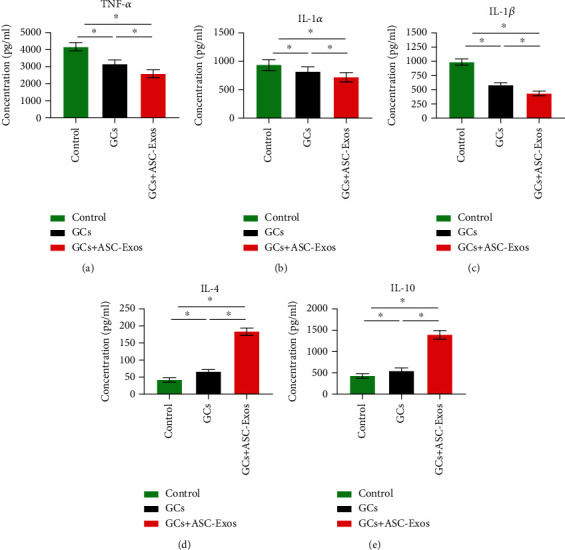
Statistical comparison of ELISA results of proinflammatory cytokines, including (a) TNF-*α*, (b) IL-1*α*, and (c) IL-1*β*, and anti-inflammatory cytokines including (d) IL-4 and (e) IL-10 secreted from rat RAW cells in the saline-treated group (control), GC-treated group (GCs), and GC+ASC-Exo-treated group (GCs + ASC-Exos). Data are expressed as mean ± SD. ^∗^indicates significant differences, *P* < 0.05.

Significant differences were also detected between the GC group and GC+ASC-Exo group, indicating that additional treatment with ASC-Exos might exert a stronger anti-inflammatory effect than dexamethasone alone (Figures 5(a)–5(c)).

### 3.5. Anti-inflammatory Cytokines

Dexamethasone treatment significantly upregulated secretion of IL-4 and IL-10 to 1.61- and 1.27-fold, respectively, compared with the control group.

Additional treatment with ASC-Exos further significantly upregulated secretion of IL-4 and IL-10 to 4.33- and 3.23-fold, respectively, compared with the control group.

Consistent with proinflammatory cytokine results, significant differences were also detected between the GC group and GC+ASC-Exo group, indicating that additional treatment with ASC-Exos might exert a stronger anti-inflammatory effect than dexamethasone alone (Figures 5(d) and 5(e)).

### 3.6. Cytoprotective Analysis

#### 3.6.1. Cell Proliferation

Dexamethasone treatment significantly decreased the proliferation intensity of rat tenocytes to 0.74-, 0.91-, and 0.70-fold at 12, 24, and 48 hours, respectively, compared with the control group.

**Figure 6:**
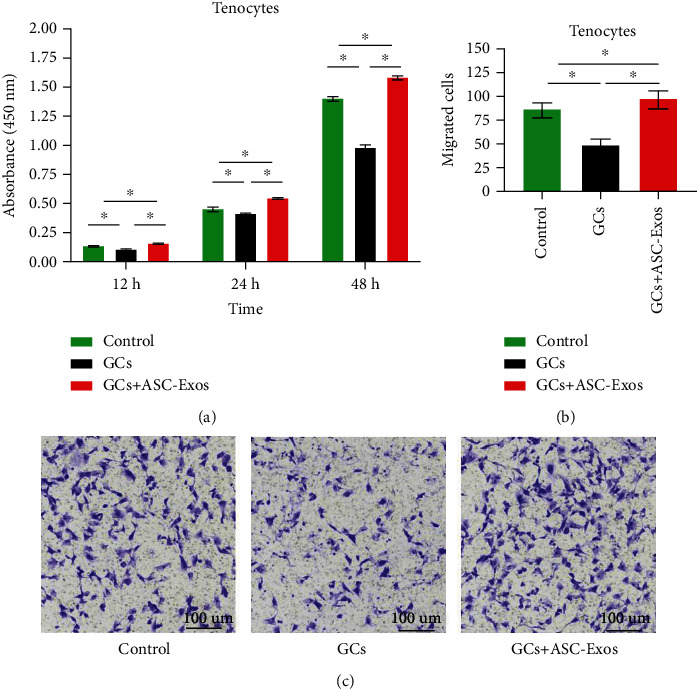
(a) Statistical comparison of rat tenocyte proliferation intensity in the saline-treated group (control), GC-treated group (GCs), and GC+ASC-Exo-treated group (GCs + ASC-Exos). (b) Statistical comparison of rat tenocytes migration activity in the saline-treated group (control), GC-treated group (GCs), and GC+ASC-Exo-treated group (GCs + ASC-Exos). (c) Representative figures of rat tenocyte migration activity in the saline-treated group (control), GC-treated group (GCs), and GC+ASC-Exo-treated group (GCs + ASC-Exos). Data are expressed as mean ± SD. ^∗^indicates significant differences, *P* < 0.05.

Additional treatment with ASC-Exos not only overrode the detrimental effect of dexamethasone on proliferation of rat tenocytes but further significantly promoted their proliferation intensity to 1.17-, 1.22-, and 1.13-fold at 12, 24, and 48 hours, respectively, compared with the control group (Figure 6(a)).

### 3.7. Cell Migration

Dexamethasone treatment significantly decreased the migration of rat tenocytes to 0.55-fold at 24 hours, compared with the control group.

Additional treatment with ASC-Exos not only overrode the detrimental effect of dexamethasone on migration of rat tenocytes but further significantly increased their migration to 1.13-fold at 24 hours, compared with the control group (Figures 6(b) and 6(c)).

### 3.8. Cell Senescence

Dexamethasone treatment significantly increased the senescence intensity of rat tenocytes to 1.92-fold at 24 hours, compared with the control group.

**Figure 7:**
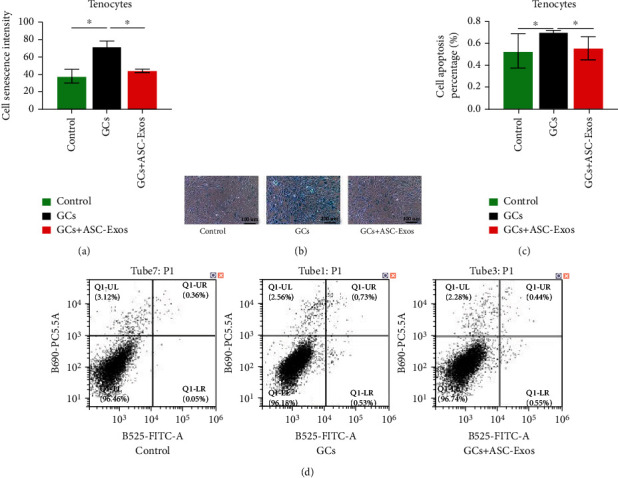
(a) Statistical comparison of rat tenocytes senescence intensity in the saline-treated group (control), GC-treated group (GCs), and GC+ASC-Exo-treated group (GCs + ASC-Exos). (b) Representative figures of *β*-galactosidase staining in the saline-treated group (control), GC-treated group (GCs), and GC+ASC-Exo-treated group (GCs + ASC-Exos). (c) Statistical comparison of rat tenocytes apoptosis percentage in the saline-treated group (control), GC-treated group (GCs), and GC+ASC-Exo-treated group (GCs + ASC-Exos). (d) Representative figures of rat tenocyte apoptosis evaluated using a flow cytometer in the saline-treated group (control), GC-treated group (GCs), and GC+ASC-Exo-treated group (GCs + ASC-Exos). Data are expressed as mean ± SD. ^∗^indicates significant differences, *P* < 0.05.

Additional treatment with ASC-Exos overrode the detrimental effect of dexamethasone on the senescence intensity of rat tenocytes, as shown by the lack of a significant difference in the senescence intensity between the GC+ASC-Exo group and the control group (Figures 7(a) and 7(b)).

### 3.9. Cell Apoptosis

Dexamethasone treatment significantly increased the apoptosis percentage of rat tenocytes to 1.34-fold at 24 hours, compared with the control group.

Additional treatment with ASC-Exos overrode the detrimental effect of dexamethasone on the apoptosis percentage of rat tenocytes, as shown by the lack of a significant difference in the apoptosis percentage between the GC+ASC-Exo group and the control group (Figures 7(c) and 7(d)).

### 3.10. Transcription of ROS

Dexamethasone treatment significantly upregulated ROS transcription of rat tenocytes to 2.40-fold at 72 hours compared with the control group.

**Figure 8:**
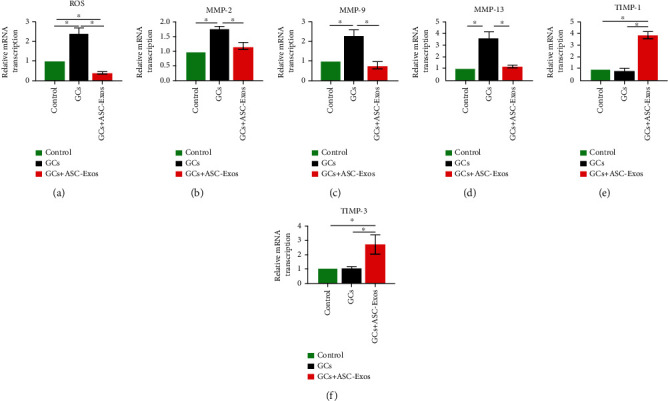
Statistical comparison of rat tenocyte PCR results of (a) ROS, degradative enzymes, including (b) MMP-2, (c) MMP-9, and (d) MMP-13, and their inhibitors including (e)TIMP-1 and (f) TIMP-3 in the saline-treated group (control), GC-treated group (GCs), and GC+ASC-Exo-treated group (GCs + ASC-Exos). Data are expressed as mean ± SD. ^∗^indicates significant differences, *P* < 0.05.

Additional treatment with ASC-Exos not only overrode the effect of dexamethasone on ROS transcription by rat tenocytes, it significantly decreased ROS transcription to 0.41-fold at 72 hours compared with the control group (Figure 8(a)).

### 3.11. Transcription of Degradative Enzymes and Their Inhibitors

Dexamethasone treatment significantly upregulated transcription of MMP-2, MMP-9, and MMP-13 by rat tenocytes to 1.79-, 2.33-, and 3.62-fold, respectively, compared with the control group. Additional treatment with ASC-Exos overrode the effect of dexamethasone on transcription of these degradative enzymes in rat tenocytes, as shown by the lack of significant difference between the GC+ASC-Exo group and the control group.

Dexamethasone treatment had little effect on transcription of degradative enzyme inhibitors by rat tenocytes, as shown by the lack of significant difference between the GC group and the control group regarding transcription of TIMP-1 and TIMP-3. However, additional treatment with ASC-Exos upregulated transcription of TIMP-1 and TIMP-3 in rat tenocytes to 3.95- and 2.72-fold at 72 hours, respectively, compared with the control group (Figures 8(b)–8(f)).

### 3.12. Transcription of Tenocytic Matrix Molecules

**Figure 9:**
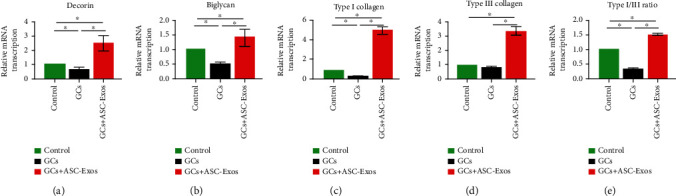
Statistical comparison of rat tenocyte PCR results of tenocytic matrix molecules including (a) decorin, (b) biglycan, (c) type I collagen, (d) type III collagen, and (e) Type I/III ratio in the saline-treated group (control), GC-treated group (GCs), and GC+ASC-Exo-treated group (GCs + ASC-Exos). Data are expressed as mean ± SD. ^∗^indicates significant differences, *P* < 0.05.

Dexamethasone treatment significantly downregulated the transcription of decorin and biglycan by rat tenocytes to 0.65- and 0.53-fold at 72 hours, respectively, compared with the control group. Additional treatment with ASC-Exos not only overrode the effect of dexamethasone on decorin and biglycan transcription by rat tenocytes, it significantly increased their transcription to 2.41- and 1.40-fold at 72 hours, respectively, compared with the control group (Figures 9(a) and 9(b)).

Dexamethasone treatment significantly downregulated transcription of type I collagen by rat tenocytes to 0.27-fold at 72 hours compared with the control group. Transcription of type III collagen was also downregulated in the GC group to 0.78-fold compared with the control group, although this difference did not reach statistical significance. Additional treatment with ASC-Exos not only overrode the effect of dexamethasone on transcription of type I and type III collagen by rat tenocytes, it significantly increased their transcription to 4.90- and 3.23-fold at 72 hours, respectively, compared with the control group (Figures 9(c) and 9(d)).

Dexamethasone treatment also significantly downregulated the type I/III transcription ratio to 0.34-fold compared with the control group. Additional treatment with ASC-Exos not only overrode the effect of dexamethasone on the type I/III transcription ratio of rat tenocytes, it further significantly increased their transcription ratio to 1.52-fold at 72 hours compared with the control group (Figure 9(e)).

### 3.13. Histological Analysis

#### 3.13.1. Injured Supraspinatus Tendon

**Figure 10:**
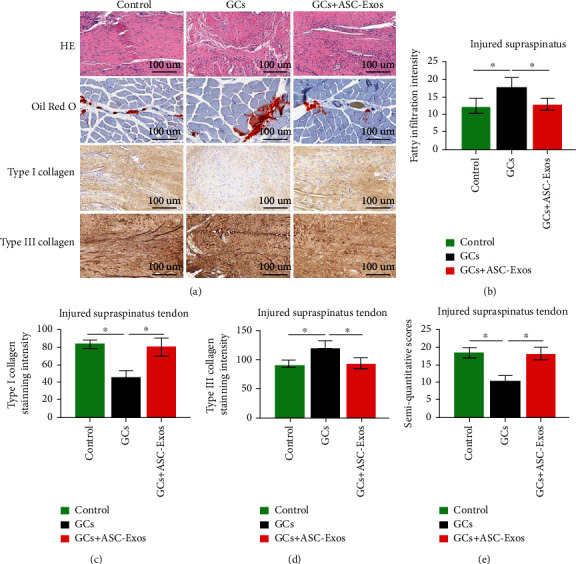
Histological analysis of the injured supraspinatus. (a) Histological staining including HE staining, oil red O staining, type I collagen staining, and type III collagen staining in the saline-treated group (control), GC-treated group (GCs), and GC+ASC-Exo-treated group (GCs + ASC-Exos). Magnification 10x. Quantitative analysis of (b) fatty infiltration intensity, (c) type I collagen staining intensity, (d) type III collagen staining intensity, and (e) semiquantitative analysis in the saline-treated group (control), GC-treated group (GCs), and GC+ASC-Exo-treated group (GCs + ASC-Exos). Data are expressed as mean ± SD. ^∗^indicates significant differences, *P* < 0.05.

Generally, increased cellularity, vascularity, thinning, separation, and disorganization of collagen fibers and fatty infiltration were observed in all three groups. These signs were most significant in the GC group (Figure 10(a)).

Quantitatively, the GC group exhibited significantly higher fatty infiltration and severe collagen degeneration (significantly lower type I collagen and higher type III collagen density) than both the control and GC+ASC-Exo groups. Immunohistochemical variables did not significantly differ between the control and GC+ASC-Exo groups (Figures 10(b)–10(d)).

Semiquantitative analysis results showed that histological properties in the GC group were significantly worse than those in the control and GC+ASC-Exo groups. There were no significant differences between the control and GC+ASC-Exo groups (Figure 10(e)).

### 3.14. Intact Infraspinatus Tendon

**Figure 11:**
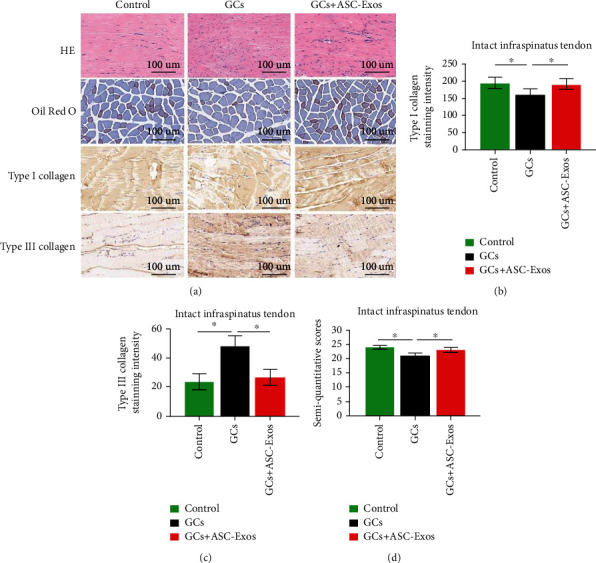
Histological analysis of the intact infraspinatus. (a) Histological staining including HE staining, Oil Red O staining, type I collagen staining, and type III collagen staining in the saline-treated group (control), GC-treated group (GCs), and GC+ASC-Exo-treated group (GCs + ASC-Exos). Magnification 10x. Quantitative analysis of (b) type I collagen staining intensity, (c) type III collagen staining intensity, and (d) semiquantitative analysis in the saline-treated group (control), GC-treated group (GCs), and GC+ASC-Exo-treated group (GCs + ASC-Exos). Data are expressed as mean ± SD. ^∗^indicates significant differences, *P* < 0.05.

Generally, slightly increased cellularity, vascularity, thinning, separation, and disorganization of collagen fibers were observed in all three groups. These signs were relatively most significant in the GC group. There was no evidence of fatty infiltration in any of the three groups (Figure 11(a)).

Quantitatively, the GC group exhibited severe collagen degeneration (significantly lower type I collagen and higher type III collagen densities) compared with the control and GC+ASC-Exo groups. There were no significant differences between the control and GC+ASC-Exo groups with regard to collagen degeneration (Figures 11(b) and 11(c)).

Semiquantitative analysis results showed that histological properties in the GC group were significantly worse than those in the control and GC+ASC-Exo groups. There were no significant differences between the control and GC+ASC-Exo groups (Figure 11(d)).

### 3.15. Biomechanical Analysis

#### 3.15.1. Injured Supraspinatus Tendon

**Figure 12:**
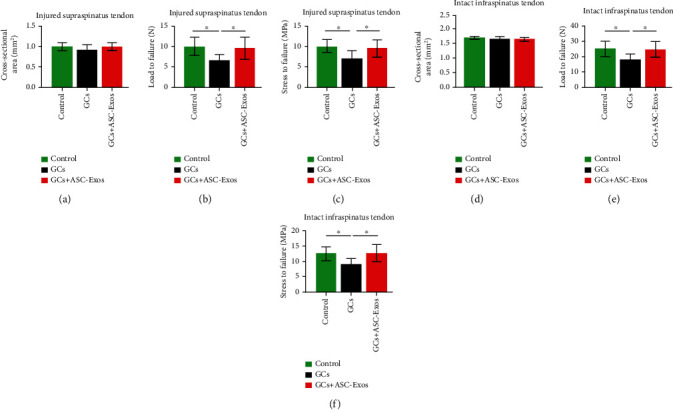
Biomechanical analysis of (a) cross-sectional area, (b) load-to-failure, and (c) stress-to-failure of injured supraspinatus tendon in the saline-treated group (control), GC-treated group (GCs), and GC+ASC-Exo-treated group (GCs + ASC-Exos) and (d) cross-sectional area, (e) load-to-failure, and (f) stress-to-failure of intact infraspinatus tendon in the saline-treated group (control), GC-treated group (GCs), and GC+ASC-Exo-treated group (GCs + ASC-Exos). Data are expressed as mean ± SD. ^∗^indicates significant differences, *P* < 0.05.

The cross-sectional area of the supraspinatus tendon did not significantly differ among the three groups (Figure 12(a)).

GC injection significantly decreased the ultimate load to failure and the ultimate stress to failure to 0.66- and 0.71-fold, respectively, compared with the control group (Figures 12(b) and 12(c)). Concomitant injection of ASC-Exos and GCs overrode GC-induced detrimental effects on biomechanical properties of the injured supraspinatus tendon, as shown by the lack of significant differences between the GC+ASC-Exo group and the control group in the ultimate load to failure and the ultimate stress to failure (Figures 12(b) and 12(c)).

All specimens failed at the middle of the tendon.

### 3.16. Intact Infraspinatus Tendon

The cross-sectional area of the intact infraspinatus tendon did not significantly differ among the three groups (Figure 12(d)).

GC injection significantly decreased the ultimate load to failure and the ultimate stress to failure to 0.64- and 0.74-fold, respectively, compared with the control group (Figures 12(e) and 12(f)). Concomitant injection of ASC-Exos and GCs overrode GC-induced detrimental effects on biomechanical properties of the intact infraspinatus tendon, as shown by the lack of significant differences between the GC+ASC-Exo group and the control group in the ultimate load to failure and the ultimate stress to failure (Figures 12(e) and 12(f)).

All specimens failed at the tendon-to-bone interface.

## 4. Discussion

**Figure 13:**
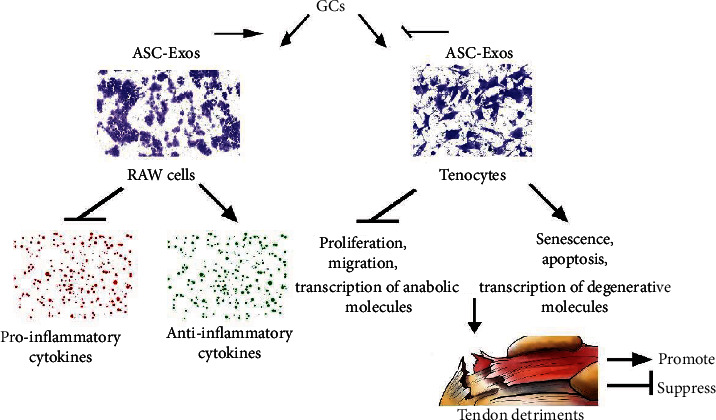
Graphical abstract. ASC-Exos exert a synergic anti-inflammatory effect with glucocorticoids and override their detrimental effects on rotator cuff tendons.

The most important finding of the present study was that additional treatment with ASC-Exos not only facilitated the anti-inflammatory effect of GCs, it overrode the negative effect of GCs on tenocytes, alleviating the detrimental effect of a GC injection on an injured-intact rotator cuff. In vitro results showed that additional treatment with ASC-Exos and GCs exerted a synergic anti-inflammatory effect mainly by suppressing secretion of proinflammatory cytokines and increasing secretion of anti-inflammatory cytokines from RAW cells. The cytoprotective effect of ASC-Exos on tenocytes was achieved mainly by suppressing GC-induced transcription of ROS and MMPs and increasing transcription of TIMPs, GC-suppressed tenocytic matrix molecules (including decorin and biglycan), and the type I/III collagen ratio. Furthermore, we established a rat model of chronic rotator cuff tear to evaluate the effect of additional treatment with ASC-Exos plus GCs on both injured and intact tendons. Results show that detriments to both the histological and mechanical properties of the injured-intact rotator cuff induced by a single GCs injection at 1 week were restored by an additional ASC-Exo injection (Figure 13).

Subacromial GC injections are commonly performed to treat multiple shoulder pathologic conditions to reduce pain and improve range of motion [3–5, 8]. However, a major concern limiting further use of GC injections is their adverse effects such as acceleration of tendon degeneration [2, 6, 7, 15, 18]. Nowadays, it is commonly accepted that subacromial GC injections lead to a significant decrease in the mechanical properties of both injured and normal rotator cuff tendons [2, 6, 15]. Inspired by the bioactive effect of platelet-rich plasma (PRP), an autologous blood plasma concentrate that contains various growth factors, Jo et al. investigated the effect of concomitant treatment with PRP and GCs on tenocytes in a degenerative condition induced by IL-1*β* [7]. Their results showed that PRP produced a synergic anti-inflammatory effect with GCs and antagonized their adverse effects [7]. In vitro evidence from other studies has also showed that some bioactive factors have a cytoprotective effect on tenocytes against GC-induced cellular senescence and apoptosis [31–33].

Macrophages play a key role during the inflammatory infiltration process after initial rotator cuff injury to cause shoulder pain, degeneration, and dysfunction [11–14, 34]. During early inflammatory infiltration, macrophages cause damage to normal tissues when phagocytizing and clearing necrotic tissues [11–13, 34]. During inflammatory regression, residual lipid-laden macrophages differentiate into adipocytes, resulting in fatty accumulation in rotator cuff muscles [35]. Furthermore, multiple proinflammatory cytokines secreted by macrophages start the inflammatory infiltration cascade that leads to the secondary damage [11–13, 34]. Thus, the main therapeutic objective to improve impaired shoulder function is to inhibit macrophage proliferation, migration, and secretion of proinflammatory cytokines. In the present study, a single GC injection significantly decreased rat RAW cell proliferation, migration, and secretion of proinflammatory cytokines including TNF-*α*, IL-1*α*, and IL-1*β* and increased secretion of the anti-inflammatory cytokine IL-4. Additional treatment with ASC-Exos plus GCs further significantly decreased secretion of proinflammatory cytokines and increased secretion of anti-inflammatory cytokines including IL-4 and IL-10, suggesting a stronger synergic anti-inflammatory effect. However, this synergic anti-inflammatory effect was not achieved by further decreasing the proliferation and migration of rat RAW cells, as the proliferation and migration intensity in the GC+ASC-Exo group were not different from the control group. We considered that this synergic anti-inflammatory phenomenon might be achieved by ASC-Exos driving transformation of macrophages to an M2 phenotype. Recent studies from Chen et al. found that medium conditioned with human bone marrow-derived stem cells, which could be regarded as exosomes from bone marrow-derived stem cells, had a significant impact on macrophage polarization by inhibiting M1 phenotype polarization and promoting M2 phenotype polarization [12]. Zhang et al. also reported that MSC-Exos enhance the survival of cartilage grafts via phenotypic transformation of macrophages from M1 to M2 [23]. These results indicate that the synergic anti-inflammatory effect obtained after additional treatment with ASC-Exos plus GCs in the present study might be achieved by regulating the phenotypic transformation of macrophages. Further studies are needed to investigate this phenomenon.

Additional treatment with ASC-Exos plus GCs not only exerted a stronger synergic anti-inflammatory effect than GC treatment alone but also restored GC-induced detrimental effects on tenocytes. ROS generation is reportedly a sensitive indicator of GC-induced mitochondrial detriments, resulting in decreased cell proliferation and migration and increased cell senescence and apoptosis [18]. In the present study, additional treatment with ACS-Exos significantly reduced the GC-induced ROS transcription, decreasing senescence and apoptosis and increasing the proliferation and migration of tenocytes. MMPs are responsible for degradation of the extracellular matrix, while this process is antagonized by TIMPs during tendon development, morphogenesis, and normal remodeling [27, 36, 37]. Although the roles of MMPs and TIMPs may be either pathological or physiological, the present results suggest that additional treatment with ASC-Exos somewhat decreased GC-induced MMP levels and increased the TIMP levels, indicating the possible role of ASC-Exos in maintaining the metabolic homeostasis of tendons [27, 36, 37]. Increased amount of type *ΙΙΙ* collagen is regarded as a repair response to tissue injury and formation of wound bed granulation tissue, while successful remodeling is characterized by the replacement of type III collagen with type I collagen in the healing of torn rotator cuff tendons [27, 36]. The present results show that GC treatment significantly decreased the type I/III collagen transcription ratio, which might result in a poorly organized tendon, while additional treatment with ASC-Exos was able to restore this adverse effect. Decorin and biglycan are also major components of leucine-rich proteoglycan in tendons [22]. Additional treatment with ASC-Exos also significantly increased GC-suppressed transcription of decorin and biglycan, further indicating that ASC-Exos had a cytoprotective effect on tenocytic metabolism.

Our hypothesis was further supported by in vivo studies showing that an additional injection of ASC-Exos significantly improved both the histological and mechanical properties of GC-induced detriments on rotator cuff tendons. Consistent with our in vitro studies, the disorganization, collagen degenerative changes from type I to type III, fatty infiltration, and compromised tendon mechanical properties induced by a GC injection were restored by an additional injection of ASC-Exos. The potential benefits underlying the contribution of ASC-Exos to synergic anti-inflammatory effects and antagonizing the detrimental effects of GCs might be attributable to stem cell-derived exosome-regulated immunity and growth effects [20, 22].

Published data concerning protection against GC-induced tendon damage are limited. Poulsen et al. investigated GC-induced detriments of tenocytes and found that vitamin C and insulin antagonize GC-induced inhibition of the ERK and Akt signaling pathways, as well as the GCs-induced increased level of FOXO, restoring normal cell proliferation and collagen synthesis in GC-treated tenocytes [18]. These previous results identify the signaling pathways underlying GC-induced detrimental effects on tenocytes, and the authors suggested potential strategies against the adverse effects of GCs.

The present study had the following limitations. First, we used normal rather than degenerative tenocytes in the in vitro study, and the results might differ under different conditions. Second, we only investigated the effect of a single GC injection with or without ASC-Exos on rotator cuff tendons. As previous studies showed that the detrimental effect of a single GCs injection on tendons is transient, both the histological and mechanical properties of the GC-treated tendon may return to normal levels by 3 weeks [2]. Third, anti-inflammatory and analgesic effects are the main areas of interest regarding GC injections in clinical practice. However, it is difficult to evaluate changes in pain levels after GC injections in an animal model, and this important aspect was lacking in the present study. Finally, the ASC-Exo concentration of 1 × 10^11^ pellets/mL used in the present study was employed according to previous studies without preexperiments to evaluate the best concentration of ASC-Exos for further investigation.

## 5. Conclusion

The combination of GCs and ASC-Exos exerted a stronger anti-inflammatory effect than GCs alone and overrode the detrimental effects of GCs on the rotator cuff.

## Figures and Tables

**Table 1:** The tendon maturing scoring system.

	1	2	3	4
Cellularity	Marked	Moderate	Mild	Minimal
Proportion of cells resembling tenocytes	<25%	25–50%	50–75%	>75%
Proportion of parallel cells	<25%	25–50%	50–75%	>75%
Vascularity	Marked	Moderate	Mild	Minimal
Proportion of fibers of large diameter characteristic of mature tendon fibers	<25%	25–50%	50–75%	>75%
Proportion of parallel fibers	<25%	25–50%	50–75%	>75%

## Data Availability

All data is available, and we are able to provide all raw data on requirement.

## Supplementary Materials

Supplementary Materials Primer sequences for mRNAs in RT-PCR analysis used in the present study are provided in the supplementary materials.
