# Supplementary material for: Anti-inflammatory and Tendon-Protective Effects of Adipose Stem Cell-Derived Exosomes with Concomitant Use of Glucocorticoids
Source: Stem Cells Int. 2022 May 20;2022:1455226. doi: 10.1155/2022/1455226 (PMC9142315; doi:10.1155/2022/1455226)
Supplement: Supplementary Materials — Primer sequences for mRNAs in RT-PCR analysis used in the present study are provided in the supplementary materials. [file 1455226.f1.docx]

**mRNAs Primer Sequences**

| Species | Gene | Direction | Sequence |
| --- | --- | --- | --- |
| Rat | ROS | FORWARD | TGCTGGTGGAGTCATCAACAAAGG |
|  |  | REVERSE | AAGGCAACTGGCATCGTGTCATC |
| Rat | Biglycan | FORWARD | GCTTCAGGCTCAGACACCACTTC |
|  |  | REVERSE | GATCTCCTTGGGCACAGTCTTCAG |
| Rat | Decorin | FORWARD | GTCATAGAACTGGGCGGCAACC |
|  |  | REVERSE | ATGTTGGTGTCTGAGATGCGGATG |
| Rat | TIMP-3 | FORWARD | GCAACTCCGACATCGTGATCCG |
|  |  | REVERSE | TAGTGTAGACCAGAGTGCCAAAGGG |
| Rat | TIMP-1 | FORWARD | CTTCCTGGTTCCCTGGCATAATCTG |
|  |  | REVERSE | TCCACAAGCAATGACTGTCACTCTC |
| Rat | MMP-13 | FORWARD | CAGCCCTATCCCTTGATGCCATTAC |
|  |  | REVERSE | GGGTGCAGACGCCAGAAGAATC |
| Rat | MMP-9 | FORWARD | CACCGCCAACTATGACCAGGATAAG |
|  |  | REVERSE | CTGCTTGCCCAGGAAGACGAAG |
| Rat | MMP-2 | FORWARD | GTGACGGCTTCCTCTGGTGTTC |
|  |  | REVERSE | CAGGGCTGTCCATCTCCATTGC |
| Rat | COLI-A1 | FORWARD | GGATGCCTCTTGTCAGTGCTAACC |
|  |  | REVERSE | TCATAGTGCTGCTGCCTGTTGTAC |
| Rat | COLIII-A1 | FORWARD | AGTCGGAGGAATGGGTGGCTATC |
|  |  | REVERSE | CAGGAGATCCAGGATGTCCAGAGG |
